# Monitoring of the Gasoline Oxygenate MTBE and BTEX Compounds in Groundwater in Catalonia (Northeast Spain)

**DOI:** 10.1100/tsw.2002.223

**Published:** 2002-05-08

**Authors:** J. Fraile, J.M. Niaerola, L. Olivella, M. Figueras, A. Ginebreda, M. Vilanova, D. Barcela

**Affiliations:** Catalan Water Agency (Agència Catalana de l'Aigua), Ministry of Environmental Affairs, Generalitat de Catalunya, Provença 204-208, 08036 Barcelona, Spain; Department of Environmental Chemistry, IIQAB-CSIC, Jordi Girona 18-26, 08034 Barcelona, Spain

**Keywords:** MTBE, BTEX, groundwater

## Abstract

Headspace (HS) gas chromatography with flame ionisation detection (HS-GC-FID) and purge and trap (P) gas chromatography-mass spectrometry (P) were used for the determination of methyl-tert-butyl ether (MTBE) and benzene, toluene, and xylenes (BTEX) in groundwater. In this work, we present the first data on the levels of MTBE and BTEX in different groundwater wells in the area of Catalonia (northeast Spain). This monitoring campaign corresponded to 28 groundwater wells that were located near petrol service stations, oil refinery storage tanks, and/or chemical industry at different locations of Catalonia during the period of 1998/1999. The levels of MTBE detected varied between 4—300 μg/l, but two sites had MTBE levels up to 3 and 13 mg/l. In many cases, the BTEX levels were below 1 μg/l, whereas 7 sites had levels varying from 19 μg/l up to 3 mg/l. Most of them were related to leakage from underground tanks in petrol service stations, while the remaining three corresponded respectively to chemical industrial pollution of undetermined origin and to a leak from high-ground petrol tanks in petrochemical refinery factories. The aquifers involved were constituted by detritus coarse materials, sands, and conglomerates. Piezometric levels were roughly comprised between 3 and 40 m, and permeability (K) and transmissivity (T) values were estimated from field measurements.

The MTBE/BTEX ratio was also calculated and reached values up to 250. These values were expected, since if we consider that spilled oxygenated gasoline is the source of well contamination and based on solubility considerations alone, the MTBE source concentrations would be about 200 times higher than any BTEX compounds.

## INTRODUCTION

Fuel oxygenates like methyl-*tert*-butyl ether (MTBE) are used as octane enhancers and decrease vehicular carbon monoxide emissions and ozone level in the atmosphere. MTBE is mainly used in the U.S. with 61% of the total use; Europe is the other relevant consumer with 15%. It was introduced in gasoline in European countries at the beginning of the 1970s. According to European regulation, the maximum concentration of MTBE in gasoline is 15 vol. %. In Spain, MTBE is used in different types of gasoline at levels between 2.8–5, 2% in 95 unleaded and up to 6.9–12.2% in 98 unleaded gasoline.

There are quite extensive reports in the U.S. about the use of MTBE, and it has been detected in groundwater wells and also lakes[1,2]. Most of the MTBE and BTEX data in the U.S. comes from utility laboratories testing drinking water as required by law. The U.S. Geological Survey plays a leading role in the monitoring of MTBE in the U.S. In Europe, different environment agencies in Denmark, the Netherlands, Switzerland, and the U.K. have initiated national studies to investigate the environmental problems associated with the intense use of MTBE[3]. Recently the Finnish Environment Institute published an extensive MTBE risk assessment study[4].

The two most common methods for determining MTBE in water are headspace (HS) and purge and trap (P&T) enrichment[5]. Basically, HS is rather suitable for highly polluted samples that can cause matrix and carry-over problems. P&T enrichment, following EPA method 624, is the most commonly used method. In order to identify hot-spot groundwater areas contaminated with MTBE and BTEX, HS will be used first for all samples. This will be of great help for determining MIBE and BTEX in highly polluted spot areas; afterwards P&T will be used for the final confirmation of HS results. This has been the analytical approach used in this paper.

In addition, the present work will fill the gap indicated in a recent overview paper that pointed out that no data on MTBE in groundwater were available for southern and eastern European countries[3]. In this work, we present the first data on the levels of MTBE and BTEX in different groundwater wells in the area of Catalonia (northeast Spain). Overall, the present work had two objectives:

- To use two methods based on HS and P&T, followed by GC-MS analysis according to EPA method 624 for MTBE and BTEX in water.
- To report the first monitoring campaign from groundwater samples in Catalonia during the period of 1998/1999. We analysed 28 groundwater wells that were located near petrol service stations, oil refinery storage tanks, and/or the chemical industry.

## EXPERIMENTAL

### Sampling, Storage, and Sample Preparation

Water samples were kept in 250-ml glass bottles filled to the top and stored at 4ºC. Samples were collected and analysed within the same week. Fig. 1 shows the different sampling points in Catalonia.

### Reagents

A standard mixture of benzene, toluene, ethylbenzene, *m*+*p*-xylene, *o*-xylene, and MTBE was prepared in methanol and added on organic-free water. α,α,α-Trifluorotoluene was used as an internal standard. Standards were obtained from SUPELCO (Barcelona).

### Analysis by Headspace (HS) GC-FID

A 10-ml water sample was sealed in a 22-ml HS vial with an open-centre aluminium cap and PTFE-faced butyl rubber septum, and analysed by HS with a FID[6]. Previously, 10 ml of 100 ppm inter-

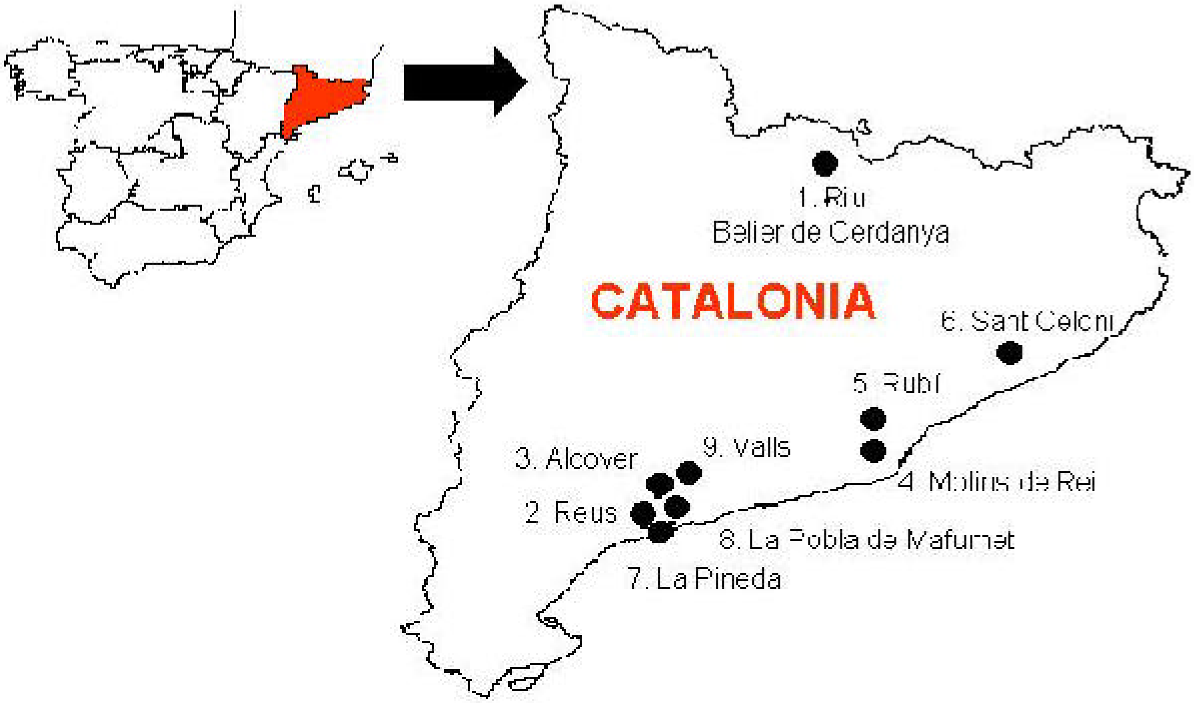

**FIGURE 1.** Map with sampling locations in Catalonia, Spain.

nal standard solution of α,α,α-trifluorotoluene was spiked. The HS analysis was performed in a Varian Genesis Headspace Autosampler connected to a Varian Star 3600 gas chromatograph. Samples were equilibrated at 70ºC for 4 min, mixed at 80% of full power for 7 min, and postmixing stabilised for 1 min. Sample loop was 1 ml; line and valve were maintained at 150ºC and vials pressurised at 7 psi.

The fused-silica column (75 m × 0.53 mm × 3 mm) was DB-624 (J&W). The GC operating temperatures were as follows: (1) injector, 160ºC; (2) detector, 300ºC; and (3) oven 40ºC (5 min) program rate at 5ºC/min until 250ºC (0 min). Helium was the carrier gas at 9 psi. Samples were quantified by HS-FID. Calibration equations were obtained for each analyte using α,α,α-trifluorotoluene as internal standard at 100 ppb. The limits of detection (LOD) varied from 0.3 to 0.5 µg/l and were calculated by using the standard deviation ($S_c$) of 7 replicates at 1 µg/l for BTEX and 1.4 µg/l for MTBE, and computed as[7]:

$$LOD = t_{(N-1,1-\alpha = 0.99)} \cdot S_c$$

SD% was obtained as the mean of 13 replicates at 3 different concentrations (1, 50, and 500 µg/l for BTEX) over all linear range.

Confirmatory analysis is recommended with MS to separate some coeluting peaks such as MTBE-acrylonitrile, benzene-isopropyl acetate, and *o*-xylene-styrene. Limits of quantitation (LOQ) varied between 1.5–2 µg/l.

The analysis of all samples at the different sites was carried out in triplicate and statistical analysis was not performed since only a limited number of samples were analysed.

## Confirmatory Analysis by P&T GC-MS

All positive samples were confirmed by P&T-MS analysis, according to EPA method 624[8]. A 20-ml sample was purged at 30ºC in a 40 ml/min helium flow and trapped on 200 mg Tenax adsorbent.

The confirmatory analysis consisted of an automated thermal desorber ATD-400 (PERKIN ELMER) connected to a FISONS gas chromatograph GC-8060 with an MS detector MD-800 via a 1-m length transfer line held at 200ºC.

Helium at 12 psi was used as carrier, desorption was performed at 300ºC for 5 min, and flows set at 54 ml/min inlet split, 12 ml/min outlet split, and 54 ml/min desorb.

The mass spectrometer in full scan utilising 70 V electron energy in the electron impact ionisation mode and scanning from 35 to 350 uma at 0.9 scan/sec was used. Source temperature was 200ºC and MS-GC interface was 210ºC.

## RESULTS AND DISCUSSION

### MTBE and BTEX Levels

Figure 1 shows the distribution of the MTBE-contaminated “hot spots” throughout the territory of Catalonia, identified along the period 1998–1999 by the Catalan Water Agency, which are currently under survey. The most relevant characteristics of the episodes and sites are reported in Table 1. Most of them are related to leakage from underground tanks in petrol service stations (Table 1, episodes #1 to #6). The remaining three correspond respectively to chemical industrial pollution of undetermined origin (episode #9) and to a leak from high-ground petrol tanks in petrochemical refinery factories (episodes #7 and #8). The aquifers involved are either semi-confined or free, with their littology constituted of detritus coarse materials, sands, and conglomerates. Piezometric levels are roughly comprised between 3 and 40 m, and permeability (K) and transmissivity (T) values have been estimated from field measurements.

In Table 2, the MTBE and BTEX levels obtained for the above-mentioned sites are reported. For most of them, more than one control is included, aiming to cover either spatial or time variations. For comparison purposes, the analytical profile includes, besides MTBE, volatile aromatics (BTEX, benzene, toluene, ethylbenzene, xylenes), since they are constituents of petrol commonly used as contamination tracers. Table 2 reports data of MTBE and BTEX in the different locations expressed in µg/l. The levels of MTBE detected varied between 4–300 µg/l, but two sites had MTBE levels up to 3 and 13 mg/l. The highest level observed corresponded to a leak from a large petrol service station facility in Reus. This leak was unusual and corresponded to a large and old petrol station facility. This episode has nothing to do with episode #1, at Riu, where there was no leak. In addition, Riu is a little village as compared to Reus and the petrol station was considerably smaller in size. High levels of MTBE were also detected in Rubi (episode #5a). In that particular case, the problem was similar to episode #1, near the city of Barcelona, where larger and older petrol station facilities are still being used.

The BTEX levels were in many cases below 1 µg/l, whereas seven sites had levels varying from 19 µg/l up to 3 mg/l. Most of them are related to leakage from underground tanks in petrol service stations, while the remaining three correspond respectively to chemical industrial pollution of undetermined origin and to a leak from high-ground petrol tanks in petrochemical refinery factories. Detritus coarse materials, sands, and conglomerates constitute the aquifers involved.

The high levels of MTBE are quite relevant. MTBE is highly water-soluble and moves nearly as fast as the groundwater itself. It is considered recalcitrant in the subsurface environment. The ability of MTBEs to travel significant distances and persist for long periods in the subsurface has important implications for public officials who have historically relied upon BTEX hydrocarbons to alert them to potential gasoline contamination. It is obvious that authorities should consider including MTBE in the routine testing of their municipal water supplies, in addition to BTEX.

From the results reported, it is clear that either HS or P&T are sufficiently sensitive to determine MTBE at levels higher than 1 µg/l. They obviously will be adequate to alert public officials in the case of the stringent primary and secondary action levels of the state of California, that were fixed at 13 and 5 µg/l, respectively.

**TABLE 1**
**Descriptive Characteristics of MTBE-Contaminated Sites**

| Town | Source of Contamination | No. of Control Wells | Aquifer | Geology | Littological Characteristics | Piezometric Level | Hydraulic Parameters |
|---|---|---|---|---|---|---|---|
| Riu | Petrol service station | 1 | Quatternary detritics and neogens of La Cerdanya | Quatternary/ Neogen | Detritic materials constituted by conglomerates, sands, and clays. Free aquifer. | 1,5–7 m | T = 1–16 $m^2$/day K = 0.5–8 m/day |
| Reus | Petrol service station | 1 | Tarragona Depression | Plioquaternary | Detritic materials constituted by conglomerates, sands, gravels, and clays Multilayer aquifer. | 12 m | T = 150 $m^2$/day[a] |
| Alcover | Petrol service station | 1 | Tarragona Depression | Plioquaternary | Detritic materials constituted by conglomerates, sands, and clays Multilayer aquifer. | 20 m | T = 150 $m^2$/day[a] |
| Molins de Rei | Petrol service station | 4 | Llobregat alluvial. Upper (surface) aquifer. | Quaternary | Detritic materials constituted by conglomerates, sands, and some clay spots. Free aquifer. | 16 m | K = 500–1,000 m/day T = 500–1,000 $m^2$/day |
| Rubí | Petrol service station | 3 | Vallès Depression | Quaternary and possible ceiling of Miocene | Slime formations intercalated with more coarse detritic materials. Free aquifer. | 9 m | K = 10–30 m/day |
| Sant Celoni | Petrol service station | 3 | Tordera alluvial | Quaternary | Detritic materials constituted by gravels and slimes. Free aquifer. | 2–3 m | K = 300 m/day T = 630 $m^2$/day |
| La Pineda | Oil refinery storage tanks | 4 | Tarragona Depression | Plioquaternary | Detritic materials constituted by conglomerates, sands, and clays. Multilayer aquifer. | 8–11 m | T = 460–330 $m^2$/day K = 48–33 m/day[a] |
| La Pobla de Mafumet | Oil refinery storage tanks | 3 | Tarragona Plioquatenary | Plioquatenary | Detritic materials constituted by conglomerates, sands, and clays. Multilayer aquifer. | 7–10 m | T = 100–500 $m^2$/day K = 1–100 m/day[a] |
| Valls | Chemical industry | 8 | Camp de Tarragona Depression | Plioquaternary | Detritic materials constituted by alluvial deposits in different directions, composed by clays and conglomerates, laterally evolving to sands. Multilayer aquifer. | 40–43 m | T = 20 $m^2$/day 80 $m^2$/day[a] |

[a] Littological local variations result in significant differences in either transmissivity (T) and/or permeability (K) values among nearby points.

## MTBE/BTEX Ratio

The MTBE/BTEX ratio was also calculated and reached values up to 250. There is much diversity of values in this ratio and it has been attributed to the persistence of MTBE and easy degradation of BTEX compounds. High levels of BTEX correspond to a recent dumping of these analytes, whereas the values of MTBE do not change much. So there is a difference in the MTBE/BTEX ratio considering the highest levels of MTBE at episodes #2a and #5a; that can be attributed to a recent leak at #5a as compared to an old problem in #2a.

These values are expected, since if we consider that spilled oxygenated gasoline is the source of well contamination and based on solubility considerations alone, the MTBE source concentrations would be about 200 times higher than any BTEX compounds. This number can be obtained considering that an oxygenated gasoline consists of 10, 5, and 2% (mole fraction basis) of MTBE, toluene,

**TABLE 2**
**Levels of MTBE and BTEX Expressed in µg/l of selected "Hot Spots" in Catalonia (Northeast of Spain)**

| Episode # well | Sampling Date | Source of Contamination | MTBE Levels | Benzene Levels | Toluene Levels | *m,p*-Xylene Levels | *o*-Xylene Levels | Ethylbenzene Levels | Ratio MTBE:Benzene |
|---|---|---|---|---|---|---|---|---|---|
| 1 | **98/12** | Petrol service station | Traces | <1 | <1 | <1 | <1 | <1 | — |
| 2 | **99/10** | Petrol service station | 13,208 | <1 | <1 | <1 | <1 | <1 | — |
| 3 | **98/03** | Petrol service station | 30 | <1 | <1 | <1 | <1 | <1 | — |
| 4a | **98/03** | Petrol service station | 89 | 7 | 4 | 4 | 2 | 25 | 13,3 |
| 4b | **98/03** | Petrol service station | 59 | 11 | 2 | <1 | <1 | <1 | 5,2 |
| 4c | **98/03** | Petrol service station | 4 | <1 | 0 | <1 | <1 | <1 | — |
| 4d | **98/03** | Petrol service station | 4 | 32 | 0 | <1 | <1 | <1 | 0,1 |
| 5a | **98/01** | Petrol service station | 3,044 | 3,678 | 30 | 278 | 429 | 2 | 0,8 |
| 5b | **98/01** | Petrol service station | 569 | 9 | <1 | <1 | <1 | <1 | 64,7 |
| 5c | **98/01** | Petrol service station | 46 | 16 | <1 | <1 | <1 | <1 | 2,8 |
| 6a | **99/10** | Petrol service station | 262 | 2 | <1 | 1 | <1 | <1 | 125,4 |
| 6b | **99/10** | Petrol service station | 12 | 2 | <1 | <1 | <1 | <1 | 6,0 |
| 6c | **99/10** | Petrol service station | 1,859 | 7 | 1 | 445 | 379 | <1 | 250,2 |
| 7a | **98/11** | Oil refinery storage tanks | 46 | 1,262 | 285 | 184 | 153 | <1 | 0,0 |
| 7a | **99/11** | Oil refinery storage tanks | 102 | 2 | <1 | 51 | 55 | <1 | 49,2 |
| 7b | **98/02** | Oil refinery storage tanks | 5 | 33 | <1 | <1 | <1 | <1 | 0,2 |
| 7b | **98/11** | Oil refinery storage tanks | 12 | 19 | <1 | <1 | <1 | <1 | 0,6 |
| 8a | **99/11** | Oil refinery storage tanks | Traces | 6 | <1 | 2 | 1 | <1 | — |
| 8b | **99/11** | Oil refinery storage tanks | 277 | 5 | <1 | <1 | <1 | <1 | 56,1 |
| 8c | **98/11** | Oil refinery storage tanks | 343 | <1 | <1 | <1 | <1 | <1 | — |
| 9a | **98/03** | Chemical industry | 14 | 19 | 453 | 19 | 6 | 16 | 0,7 |
| 9a | **99/11** | Chemical industry | Presence | 45 | 1,621 | 190 | 31 | 59 | — |
| 9b | **98/11** | Chemical industry | 33 | 2 | <1 | <1 | <1 | <1 | 20,8 |
| 9b | **99/09** | Chemical industry | 13 | 1 | <1 | <1 | <1 | <1 | 11,4 |
| 9b | **99/10** | Chemical industry | 13 | 2 | 1 | 1 | <1 | <1 | 5,4 |
| 9c | **99/10** | Chemical industry | 3 | <1 | <1 | <1 | <1 | <1 | — |
| 9d | **98/03** | Chemical industry | 73 | 36 | 1,029 | 26 | 4 | 13 | 2,0 |
| 9e | **99/05** | Chemical industry | Presence | 58 | 1,381 | 56 | 8 | 26 | — |

and benzene, respectively, and then the ratios of concentrations in water equilibrated with the gasoline would be 270/1.4/1.

## COMPARISON WITH OTHER E.U. COUNTRIES

The most comprehensive study until now on the E.U. level of MTBE in groundwater wells has been published by Schmidt[3]. In this review, maximum levels of MTBE varied from 120 µg/l in the

Netherlands, up to 830 mg/l in the U.K. caused by leaks in gasoline stations. In this review article, Denmark, France, Germany, and Switzerland also report values within these ranges. The values reported in Catalonia are also within the E.U. ranges. The maximum value detected in this first monitoring was 13 mg/l, which is a bit lower than the highest episodes detected in the U.K., Denmark, or Germany. Generally, the range of our values varied between 4–300 µg/l. Obviously this is a problem, especially when such groundwater needs to be used as drinking water.

## CONCLUSIONS

HS GC-FID can be used for routine monitoring of MTBE and BTEX in groundwater influenced by point source pollution. P&T GC-MS has been used as confirmatory purposes, with previous dilution of the sample when the concentration levels of the spill are too high.

From the data reported, it is still too early to evaluate the risk of MTBE in groundwater resources. As we indicated before, this is the first report of MTBE contamination in southern Europe, and there is still a lack of data in many E.U. countries. It is clear that the reported values indicate that MTBE contamination of groundwater can be a European problem and that, although more monitoring is needed to know the extent of this contamination, some actions need to be taken by the authorities in this respect. Future monitoring programs of volatile organic compounds in groundwater should also include MTBE and related compounds. In this respect, the Commission of the European Communities has started some actions. In a recent E.U. document[9], the first conclusions of the risk associated with MTBE were pointed out. It was mentioned that concern for the potability of groundwater with respect to taste and odour as a consequence of exposure arising from leaking underground storage tanks and spillage from overfilling tanks should be considered. In view of that, it was indicated that prevention of contamination of groundwater by MTBE should be a key objective of future E.C. legislation. It is recommended, therefore, that monitoring programmes be undertaken, where appropriate, to permit the early detection of groundwater contamination by MTBE. Obviously, the hot-spot areas of this first monitoring campaign undertaken in Catalonia together with the Catalan Water Authority are a starting point to tackle the problem of MTBE contamination of groundwater.

Future work is in progress by monitoring the wells indicated in this work and further wells spread over the Catalan territory. In addition, a method is being developed that will permit researchers to determine other fuel oxygenates like ethyl *tert*-butyl ether (ETBE), di-isopropyl ether (DIPE), *tert*-amyl methyl ether (TAME), *tert*-butyl formate (TBF), and *tert*-amyl methyl acohol (TAA). The new method will be used for routine monitoring of MTBE and related fuel oxygenates in our current monitoring programmes.

## ACKNOWLEDGEMENTS

This research is part of the WATCH (EVK1-CT-2000-00059) project that is being funded by the E.U. Environment and Sustainable Development subprogram and from the Ministerio de Ciencia y Tecnologia (REN2001-5039-CE).
